# The LELEX initiative to enhance cancer imaging communication: development of a consensus-based structured reporting template and lexicon for general oncologic imaging

**DOI:** 10.1590/0100-3984.2025.0088-en

**Published:** 2026-02-26

**Authors:** Natally Horvat, Fabio Ynoe Moraes, Lucia Antunes Chagas, Vanessa Freitas Bratti, Adriano Tachibana, Almir Galvão Vieira Bitencourt, Bernardo Peres Salvajoli, Camila Trolez Amancio, Carlos Augusto dos Anjos, Douglas Jorge Racy, Fernando Morbeck Coelho, Giuseppe D’Ippolito, Graziela Campos Oliveira, Joao V. Horvat, Jose de Arimateia Araujo-Filho, Leandro Machado Colli, Luciana Holtz de C. Barros, Luis Filipe de Souza Godoy, Luiz Paulo Kowalski, Marcelo Araujo Queiroz, Márcio Valente Yamada Sawamura, Maria Clara Fernandes, Marisa Riscalla Madi, Maria Ignez Braghiroli, Paulo Victor Partezani Helito, Richard Kinh Gian Do, Rodrigo Sampaio Chiarantano, Rodrigo Pamplona Polizio, Rubens Chojniak, Ulysses Ribeiro Junior, Valdair Francisco Muglia, Paulo Marcelo Gehm Hoff, Carlos Alberto Buchpiguel, Giovanni Guido Cerri, Hedvig Hricak, Cesar Higa Nomura

**Affiliations:** 1 Department of Radiology, Mayo Clinic, Rochester, MN, USA.; 2 Departments of Oncology and Medicine, Queen’s University, Kingston, ON, Canada.; 3 Department of Radiology, University of São Paulo School of Medicine, São Paulo, SP, Brazil.; 4 Department of Radiology, Hospital Israelita Albert Einstein, São Paulo, SP, Brazil.; 5 Department of Radiology, A.C. Camargo Cancer Center, São Paulo, SP, Brazil.; 6 Department of Radiation Oncology, University of São Paulo School of Medicine, São Paulo, SP, Brazil.; 7 Department of Radiology, Hospital Sirio-Libanes, São Paulo, SP, Brazil.; 8 Department of Radiology, Beneficência Portuguesa de São Paulo, São Paulo, SP, Brazil.; 9 Department of Radiology, School of Medicine, Universidade Federal de São Paulo, São Paulo, SP, Brazil.; 10 Department of Oncology, University of São Paulo School of Medicine, São Paulo, SP, Brazil.; 11 Oncoguia Institute, São Paulo, SP, Brazil.; 12 Department of Oncology, A.C. Camargo Cancer Center, São Paulo, SP, Brazil.; 13 Department of Radiology, Memorial Sloan Kettering Cancer Center, New York, NY, USA.; 14 Department of Radiology, Aspetar, Doha, Qatar.; 15 Department of Radiology, Hospital do Cancer de Barretos, São Paulo, SP, Brazil.; 16 Department of Gastroenterology and Nutrology, University of São Paulo School of Medicine, São Paulo, SP, Brazil.

**Keywords:** Radiology information systems, Radiology/education, Medical records/standards, Terminology as topic, Neoplasms/diagnostic imaging, Medical oncology/education, Decision making, Uncertainty, Longitudinal studies, Follow-up studies, Sistemas de informação em radiologia, Prontuários médicos/normas, Terminologia como assunto, Neoplasias/diagnóstico por imagem, Oncologia/educação, Tomada de decisões, Incerteza, Estudos longitudinais, Seguimentos

## Abstract

**Objective:**

The Laudo Estruturado e Léxico em Oncologia (LELEX, Lexicon and Structured Reporting in Oncology) initiative was established to develop a standardized lexicon and structured reporting system applicable across cancer types.

**Methods:**

The São Paulo Radiology Society and Brazilian Society of Medical Oncology assembled a 27-member multidisciplinary consensus panel that included radiologists, medical and radiation oncologists, surgeons, and a patient representative. A literature review identified relevant oncologic reporting frameworks to guide the creation of a draft template and lexicon by the LELEX executive committee. Panelists were surveyed to assess current reporting practices and preferences between free-text and structured reports. Consensus (i.e., agreement by more than 80% of panelists) on the final lexicon and reporting system was reached through post-survey panel discussions.

**Results:**

Compared with a traditional free-text report, a structured report incorporating the LELEX lexicon received significantly higher ratings for precision and was deemed better for guiding clinical decisions by > 88% of panelists. In post-survey discussions, consensus was achieved for all lexicon components, including diagnostic certainty terms, lesion count descriptors, and longitudinal comparison language. The structured report template was also agreed upon by the panel members.

**Conclusion:**

The LELEX initiative established a consensus-driven structured reporting system and lexicon for general oncologic imaging baseline and follow-up assessments.

## Abstract (pt)

**Objetivo:**

A iniciativa Laudo Estruturado e Léxico em Oncologia (LELEX) foi criada para desenvolver um léxico padronizado e um sistema de laudo estruturado aplicável a diferentes tipos de câncer.

**Métodos:**

A Sociedade Paulista de Radiologia e a Sociedade Brasileira de Oncologia Clínica reuniram um painel multidisciplinar de consenso composto por 27 membros, incluindo radiologistas, oncologistas clínicos e radioterápicos, cirurgiões e um representante de pacientes. Uma revisão da literatura identificou estruturas de laudos oncológicos relevantes para orientar a criação de um modelo preliminar e de um léxico pelo comitê executivo do LELEX. Os painelistas foram pesquisados para avaliar as práticas atuais de elaboração de laudos e as preferências entre laudos em texto livre e estruturados. O consenso (isto é, concordância de mais de 80% dos painelistas) sobre o léxico e o sistema de laudos finais foi alcançado por meio de discussões do painel após a pesquisa.

**Resultados:**

Em comparação com um laudo tradicional em texto livre, um laudo estruturado incorporando o léxico LELEX recebeu avaliações significativamente mais altas quanto à precisão e foi considerado melhor para orientar decisões clínicas por mais de 88% dos painelistas. Nas discussões pós-pesquisa, obteve-se consenso para todos os componentes do léxico, incluindo termos de certeza diagnóstica, descritores de contagem de lesões e linguagem de comparação longitudinal. O modelo de laudo estruturado também foi aprovado pelos membros do painel.

**Conclusão:**

A iniciativa LELEX estabeleceu um sistema de laudo estruturado e um léxico para avaliações iniciais e de seguimento em imagem oncológica geral, fundamentados no consenso.

## INTRODUCTION

Radiological imaging plays essential roles in the diagnosis, staging, treatment response assessment, and follow-up of patients with cancer. However, the lack of consistency in the structure and terminology of traditional free-text reports can make such reports difficult for referring physicians and patients to interpret^**(^1,2^)**^. In turn, this inconsistency can compromise clinical decision-making^**(^3^)**^ and weaken the potential of radiological data to facilitate precision oncology.

Cancer represents a growing global health crisis due to its relentless rise in incidence and its profound impact on health systems worldwide. According to data from the World Health Organization (WHO), the number of new cancer cases worldwide has nearly doubled over the past two decades—from 10 million in 2000 to 19.3 million in 2020. Alarmingly, the actual number in 2020 far exceeded the number that had been predicted (around 15 million). Looking ahead, the burden is projected to increase even further, with 28.4 million new cancer cases expected in 2040, marking a 47% increase over 2020^**(^4^)**^. These escalating numbers highlight the urgent need for more effective strategies in cancer prevention, diagnosis, and care, as well as the importance of optimizing the use of imaging in supporting timely and accurate clinical decision-making. Several efforts have been made to improve radiology communication through the adoption of structured reporting. Studies have shown that standardization of reporting through consistency of layout and vocabulary enhances clarity, improves satisfaction among referring physicians and facilitates utilization in clinical practice, which in turn optimizes clinical workflows^**(^5,6^)**^. Structured reporting systems and standardized lexicons have been particularly effective in ensuring that key imaging findings are consistently conveyed. Research comparing structured reporting and free-text formats has demonstrated that structured reporting, particularly when linked to specific oncologic scenarios, can increase the value of reports as perceived by oncologists and other stakeholders^**(^1,3^)**^. While the benefits are well documented, the adoption of structured reporting and standardized lexicons in oncology remains limited, particularly in complex, multidisciplinary environments. Although several standardized templates have been developed for specific oncologic scenarios—such as the Breast Imaging Reporting and Data System (BI-RADS) for breast cancer^**(^7^)**^, the Liver Imaging Reporting and Data System (LI-RADS) for hepatocellular carcinoma^**(^8^)**^, as well as dedicated templates and lexicons for rectal^**(^9^)**^ and prostate^**(^10^)**^ cancers, including the Prostate Imaging Reporting and Data System (PI-RADS)—these systems are typically confined to a single tumor type or organ system. The Response Evaluation Criteria in Solid Tumors (RECIST) guidelines^**(^11^)**^ offer a structured approach for assessing tumor response. However, because of complexities in implementation and their limited applicability to real-world clinical workflows^**(^12^)**^, their use remains largely limited to clinical trials and research environments. There is a notable gap in the literature regarding comprehensive oncologic lexicons that can be applied across cancer types and imaging modalities to support broader communication in multidisciplinary cancer care. In this setting, the *Laudo Estruturado e Léxico em Oncologia *(LELEX, Lexicon and Structured Reporting in Oncology) initiative was developed to improve multidisciplinary communication of imaging findings. Specifically, the initiative aimed to develop a consensus-based standardized lexicon and structured radiology reporting template for general oncologic imaging—applicable across all cancer types and imaging modalities—to enhance communication, reduce ambiguity, and support multidisciplinary cancer care. This collaborative initiative, led by the São Paulo Radiology Society and the Brazilian Society of Medical Oncology, brought together a multidisciplinary panel of radiologists, oncologists, surgeons, and a patient representative. Through a systematic literature review, expert consensus process, and comparative surveys regarding free-text versus structured reports, the initiative sought to establish a clear, clinically relevant framework adaptable to routine oncology practice.

## MATERIAL AND METHODS

### Panel selection

The São Paulo Radiology Society, in collaboration with the Brazilian Society of Medical Oncology, launched the LELEX initiative. This collaborative effort was led by the executive committee (C.H.N., H.H., P.M.G.H., N.H., F.Y.M., and C.A.B.), which appointed C.H.N., a radiologist, as chair. Two vice-chairs were also designated: H.H., representing radiology; and P.M.G.H., representing medical oncology. The executive committee assembled the LELEX multidisciplinary consensus panel, composed of radiologists focused on oncologic imaging, experts from various oncology subspecialties, and a patient advocacy representative.

Specifically, the LELEX consensus panel included abdominal radiologists (A.T., D.J.R., F.M.C., G.C.O., G.D., L.A.C., M.C.F., R.P.P., R.C., and V.F.M.); thoracic and cardiothoracic radiologists (J.A.A.F., M.V.Y.S., R.C., and R.S.C.); nuclear medicine radiologists (C.A.B. and M.A.Q.); a musculoskeletal radiologist (P.V.P.H.); neuroradiologists (C.T.A. and L.F.S.G.); breast radiologists (A.G.V.B. and J.V.H.); a general radiologist (M.R.M.); radiation oncologists (B.P.S. and F.Y.M.); medical oncologists (C.H.A., L.M.C., M.I.B., and P.M.G.H.); surgeons (L.P.K. and U.R.J.); and a nationally and internationally recognized patient advocacy representative (L.H.C.B.), who is a psycho-oncologist with over two decades of experience leading hospitaland policy-level initiatives across all cancer types and founded a nonprofit organization dedicated to promoting education, support, and health equity for cancer patients in Brazil and globally. An earlyto mid-career radiologist (L.A.C.) with expertise in oncologic imaging was appointed to lead a systematic review of the literature, aiming to establish the current state of evidence. The LELEX consensus panel members came from diverse clinical backgrounds and included internationally recognized experts in the field of oncology, who contributed national and global perspectives. Members were selected based on their expertise, leadership roles, and contributions to peer-reviewed literature to ensure broad representation across various aspects of oncologic patient care.

### Stepwise process

#### Literature review

One of the authors (L.A.C.) conducted a systematic literature review using the US National Library of Medicine PubMed database. The search was performed in February 2023 and focused on peer-reviewed articles related to radiology reporting, structured templates, and oncologic imaging communication. The initial search yielded 19 published works, including 13 original articles, three review articles, and three editorials.

On the basis of the findings of the literature review, the LELEX executive committee identified two key works that aligned closely with the goals of the initiative: the Certainty Lexicon, proposed in Panicek and Hricak^**(^13^)**^, and the Oncologic Response Lexicon, outlined in Do et al.^**(^14^)**^. Given the relevance and demonstrated impact of those two key works, the executive committee elected to focus its discussions and decisions around the strategies proposed therein while ensuring their adaptation to local clinical and linguistic contexts.

#### Initial multidisciplinary consensus panel survey

Initially, two separate surveys created by the executive committee were administered to two target groups: non-radiologist and radiologist members of the multidisciplinary consensus panel. The surveys assessed the panelists’ perceptions regarding their clinical activities, the frequency with which they read radiological reports in an oncologic setting, radiology report clarity, radiology report precision, and radiology report usefulness. Each survey included two anonymized computed tomography reports for the same patient. One of these reports was in a traditional free-text format (Report 1, Supplement 1); the other was in a structured format incorporating a standardized lexicon (Report 2, Supplement 2), aligned with the approaches proposed by Panicek and Hricak^**(^13^)**^ and Do et al.^**(^14^)**^. The surveys were developed and then distributed online via a secure platform. Panelists were asked to compare the two radiology report formats across key parameters—clarity, diagnostic precision, and satisfaction—as well as to indicate which report better supported clinical decision-making. The radiologist survey included additional questions regarding training background, subspecialty, practice setting, and experience with oncologic imaging. Both surveys incorporated quantitative Likert-scale questions and optional open-ended questions for qualitative feedback and suggestions. Data were collected anonymously, and responses were reviewed to inform the discussions of the consensus panel, which revised the structured reporting template and lexicon used in the survey to develop the LELEX standardized reporting template and lexicon.

**Supplement 1.** Report 1 (presented in Portuguese and English) corresponds to the traditional free-text format used in the LELEX survey. It was provided to committee members for review and evaluation as part of the comparative analysis.

Portuguese	English
**RESSONÂNCIA MAGNÉTICA DO ABDOME SUPERIOR COM COLANGIORRESSONÂNCIA**	**MAGNETIC RESONANCE CHOLANGIOPANCREATOGRAPHY**.
**Indicação:** Câncer de reto com metástases hepáticas.	**Indication:** Rectal cancer with liver metastases.
**Técnica:** Realizadas aquisições multiplanares, ponderadas em T1, em fase e fora de fase, em T2 e difusão, antes e após a administração do contraste venoso. Foram realizadas imagens fortemente pesadas em T2 para estudo colangiográfico.	**Technique:** Multiplanar acquisitions performed with T1-weighting in-phase and out-of-phase, T2-weighting, and diffusion, before and after intravenous contrast administration. Heavily T2-weighted images were obtained for cholangiographic evaluation.
**Análise:**	**Analysis:**
Análise comparativa com RM do abdome superior realizada no dia 22/08/2022	Comparative analysis with MRI from 08/22/2022
Houve discreto aumento da ascite, hoje em moderada quantidade.	There was a slight increase in ascites, now of a moderate amount.
Surgiu tecido de limites mal definidos ocupando os espaços adiposos do hilo hepático em topografia do terço proximal do hepatocolédoco, determinando redução abrupta de calibre do mesmo e acentuação da ectasia das vias biliares intra e extra-hepáticas, bem como redução da amplitude luminal do terço proximal da veia porta.	Ill-defined tissue appeared occupying the fat planes of the hepatic hilum at the level of the proximal third of the common bile duct, causing an abrupt reduction in caliber and marked ectasia of the intraand extra-hepatic bile ducts, as well as reduced luminal diameter of the proximal third of the portal vein.
Permanecem um pouco mais evidente do que nos estudos anteriores as lesões no segmento 4 junto à fissura do ligamento falciforme e área de espessamento tecidual hilar alta que determina redução abrupta de calibre em topografia da confluência biliar, sendo mais importante no ducto hepático esquerdo, e condiciona acentuação da dilatação biliar a montante, previamente individualizada, bem como afilamento do ramo esquerdo da porta e de ramo regional da artéria hepática esquerda.	The lesions in segment 4 near the falciform ligament fissure and the area of high hilar tissue thickening are slightly more evident than in previous studies, causing abrupt narrowing at the biliary confluence, predominantly affecting the left hepatic duct, leading to accentuated upstream biliary dilatation, previously noted, as well as tapering of the left portal vein branch and regional branch of the left hepatic artery.
Permanece espessamento tecidual envolvendo em 50% o tronco celíaco distal que encarcera a artéria hepática comum (que apresenta irregularidade parietal) e a origem das hepáticas direita e esquerda, notadamente esta última, sem evidente estenose, já relatado previamente.	Tissue thickening persists, involving 50% of the distal celiac trunk, encasing the common hepatic artery (which shows wall irregularity) and the origins of the right and left hepatic arteries, especially the latter, without evident stenosis, as previously reported.
Há extensão do referido espessamento tecidual à região anterior à crura diafragmática direita, lateralmente à origem da artéria mesentérica superior.	The aforementioned tissue thickening extends to the region anterior to the right diaphragmatic crus and laterally to the origin of the superior mesenteric artery.
Persistem inalteradas em número e em tamanho os nódulos hipovasculares secundários, definindose como lesões alvo as mais conspícuas: aglomeradas junto a superfície diafragmática do segmento 8 medindo em conjunto 8,2 × 4,3 cm inalterada. - no segmento 3, medindo 5,8 × 4,5 cm estável. - no segmento 6, medindo 4,8 × 4.1 cm inalterada.	The secondary hypovascular nodules remain unchanged in number and size, with the most conspicuous ones defined as target lesions: - clustered near the diaphragmatic surface of segment 8, collectively measuring 8.2 × 4.3 cm, unchanged. - in segment 3, measuring 5.8 × 4.5 cm, stable.- in segment 4, measuring 4.8 × 4.1 cm, unchanged.
Linfonodos proeminente à esquerda da origem da artéria mesentérica superior (1,6 × 1,0 cm) e no hilo hepático (2,5 × 1,2 cm).	Prominent lymph nodes to the left of the origin of the superior mesenteric artery (1.6 × 1.0 cm) and in the hepatic hilum (2.5 × 1.2 cm).
Persiste de aspecto semelhante a circulação colateral intracavitária, sendo mais importante periesplênica, associada a shunt esplenorrenal. Há ainda importante circulação colateral na tela subcutânea superficial, persistindo os sinais de má distribuição hídrica.	Intracavitary collateral circulation remains similar in appearance, most notably perisplenic, associated with a splenorenal shunt. There is also significant collateral circulation in the superficial subcutaneous tissue, with persistent signs of abnormal fluid distribution.
Esplenomegalia homogênea, medindo cerca de 15,4 cm no maior eixo coronal.	Homogeneous splenomegaly, measuring approximately 15.4 cm on the longest axis in the coronal plane.
Adrenais anatômicas.	Adrenal glands are unremarkable.
Achado de relevância não-oncológica:	Relevant non-oncologic finding:
Rins tópicos, com forma e volume normais.	Kidneys in normal location, with normal shape and volume.
Ausência de dilatação dos sistemas pielocalicianos.	No dilatation of the renal pelvis.
Aorta de curso e calibre normais.	Aorta with a normal course and caliber.

**Supplement 2.** Report 2 (presented in Portuguese and English) corresponds to the structured report with standardized lexicon provided to LELEX committee members for review and evaluation as part of the comparative analysis. The case is from the same anonymized patient and imaging study described in Report 1 (shown in Supplement 1).

Portuguese	English
**RESSONÂNCIA MAGNÉTICA DO ABDOME SUPERIOR COM COLANGIORRESSONÂNCIA**	**MAGNETIC RESONANCE CHOLANGIOPANCREATOGRAPHY**.
DADOS CLÍNICOS: Câncer de reto com metástases hepáticas.	CLINICAL INFORMATION: Rectal cancer with liver metastases.
TÉCNICA: Realizadas aquisições multiplanares antes e após a administração venosa meio de contraste hepatobiliar.	TECHNIQUE: Multiplanar acquisitions were performed before and after administration of hepatobiliary contrast.
COMPARAÇÃO: RM 22/08/2022	COMPARISON: MR from 08/22/2022
ACHADOS:	FINDINGS:
Hepatobiliar: Nova lesão irregular no hilo hepático, suspeito para metástase, infiltrando colédoco e veia porta.	Hepatobiliary: New irregular lesion at the hepatic hilum, suspicious for metastasis, infiltrating the common bile duct and portal vein.
Inalteradas as múltiplas metástases hepáticas bilobares, por exemplo: -Segmento 8, confluentes, 8.2 × 4,3 cm. -Segmento 6, 4,8 × 4,1 cm.	Unchanged multiple bilobar hepatic metastases, for example: - Segment 8, confluent lesions, 8.2 × 4.3 cm - Segment 6, 4.8 × 4.1 cm
Aumento da moderada dilatação das vias biliares, até o plano do hilo hepático devido tecido irregular.	Increased moderate biliary ductal dilation, up to the level of the hepatic hilum, due to the irregular tissue.
Baço: Esplenomegalia. Varizes periesplênica e shunt esplenorrenal.	Spleen: Splenomegaly. Perisplenic varices and splenorenal shunt.
Pâncreas: Sem alterações relevantes.	Pancreas: Unremarkable.
Adrenais: Sem alterações relevantes.	Adrenals: Unremarkable.
Rins: Sem alterações relevantes.	Kidneys: Unremarkable.
Linfonodos: Inalterados poucos linfonodos abdominais, com morfologia sugestiva de metástases: • À esquerda da origem da artéria mesentérica superior, 1,6 × 1,0 cm • Hilo hepático, 2,5 × 1,2 cm	Lymph nodes: Unchanged few abdominal lymph nodes, with morphology suggestive of metastases: • To the left of the origin of the superior mesenteric artery, 1.6 × 1.0 cm • Hepatic hilum, 2.5 × 1.2 cm
Peritônio/mesentério/intestino: Aumento da ascite, atualmente moderada.	Peritoneum/mesentery/bowel: Increased ascites, now moderate.
Ossos / partes moles: Circulação colateral e edema subcutâneo.	Bones / soft tissues: Subcutaneous collaterals and edema.
Outros: Inalterado tecido retroperitoneal metastático infiltrando aorta, tronco celíaco e crura diafragmática direita.	Other: Unchanged metastatic retroperitoneal tissue infiltrating the aorta, celiac trunk, and right diaphragmatic crus.
Impressão:	Impression:
Em comparação com TC 08/12/2022: 1. Inalteradas as múltiplas metástases hepáticas. 2. Nova lesão irregular no hilo hepático, causando aumento da moderada dilatação das vias biliares, consistente com metástase. 3. Inalteradas as poucas metástases linfonodais e retroperitoneal. 4. Aumento da ascite, atualmente moderada.	Since CT 12/08/2022: 1. Unchanged multiple hepatic metastases. 2. New irregular lesion at the hepatic hilum causing increased moderate biliary ductal dilation, consistent with metastasis. 3. Unchanged few lymph node and retroperitoneal metastases. 4. Increased ascites, now moderate.

#### Lexicon and standardized report general oncologic imaging assessment

The guidance and standardization proposed in Panicek and Hricak^**(^13^)**^, along with the Oncologic Response Lexicon provided in Do et al.^**(^14^)**^, were adapted and translated by the executive committee. The translated lexicon and a proposed standardized reporting template were reviewed and refined through two rounds of online meetings with members of the multidisciplinary consensus panel. A follow-up survey was then distributed to all panel members, listing the components of the standardized report and lexicon for approval. Consensus was defined as agreement by more than 80% of members and was reached for all core components of the lexicon and reporting system. As detailed in Table 1, the lexicon core components include the following: certainty terms aligned with estimated probabilities; standardized terminology for estimating the lesion count; comparison terms to support subjective assessment of temporal change; and guidance on reporting representative lesions per organ. A standardized report format, created by the executive committee and specifically designed for baseline and follow-up assessments of chest, abdominal, and pelvic scans, was reviewed and approved by the consensus panel to ensure the inclusion of predefined sections for clinical data, imaging technique, structured findings organized by anatomical region, and an impression summarizing relevant changes.

**Table 1:** LELEX lexicon core components and structured reporting system.

Component	Description	Consensus recommendation
Certainty lexicon*	Standardized terms linked to estimated probabilities to express diagnostic confidence	*“Consistent with”: *estimated probability > 90% “*Suggestive of*”: estimated probability ~ 75% “*Possible*”: estimated probability ~ 50% “*Less likely*”: estimated probability < 25%
Lesion counting lexicon	Terms used to estimate the number of lesions in a consistent manner	“*Few*”: 1–5 lesions “*Multiple*”: ≥ 6 lesions
Comparison terminology^†^	Subjective assessment of change over time using standardized language	“*New*”: Finding not seen on prior imaging “*Increased*”^‡^: Significant enlargement compared with prior imaging “*Slightly increased*”: Minimal enlargement compared with prior imaging “*Unchanged*”: No appreciable difference from prior imaging “*Slightly decreased*”: Minimal reduction in size “*Decreased*”: Significant reduction in size “*Markedly decreased”*: Substantial reduction in size “*Resolved*”: Finding no longer present on current imaging For each site of disease, describe two representative lesions in list format, including their location, size (in two orthogonal axial dimensions), and prior measurements, if changed.
Representative lesions	Guidance to report two representative lesions per organ, with location, size, and previous measurements, if changed	Example 1: A few increased liver metastases, for example: Segment 2, 2.5 × 2.0 cm, previously 1.5 × 1.0 cm. Segment 5, 1.8 × 1.0 cm, previously 1.0 × 0.5 cm. Example 2: Unchanged multiple peritoneal implants, for example: Right paracolic gutter, 2.5 × 1.5 cm. Left omental, 1.5 × 1.0 cm.
Structured reporting system	Key elements to include in a general oncologic imaging report	Imaging modality, body region, and date of the exam Clinical information Technique—Include details on intravenous contrast, oral contrast, acquisition method, and post-processing Comparison—Specify the modality and date of the prior imaging study used for direct comparison. Findings (organ-by-organ checklist)—When no relevant abnormalities are present, use concise terms such as “unremarkable” or “normal”. Clearly benign incidental findings may be briefly described at the radiologist’s discretion. If included, keep the description succinct (e.g., “simple renal cyst”) and avoid unnecessary detail. Impression—Provide a concise summary of key findings, including temporal context (comparison with prior imaging) Example: Since CT 02/02/2025: - Increased multiple liver metastases. - Increased peritoneal carcinomatosis.

CT, computed tomography.

* If an imaging finding is clearly diagnostic—such as a cyst, hemangioma, or known metastasis—the use of the certainty lexicon may be omitted. For example: Unchanged multiple liver metastases.

† Clearly benign lesions do not require comparison with prior studies; a brief description is sufficient—for example, “Renal cysts.”

‡ Use of the term “Markedly increased” is not recommended.

### Statistical analysis

Categorical variables are expressed as absolute frequencies and percentages. Where appropriate, continuous variables are summarized using means, standard deviations, and ranges. To evaluate differences in radiologists’ perceptions between Report 1 and Report 2, categorical data were compared by using the chi-square test. Response frequencies for clarity, precision, and satisfaction were analyzed independently.

To ensure the validity of the chi-square test, response categories with sparse or zero frequencies were grouped into broader, conceptually similar categories. For example, “Very clear” and “Clear” were combined into a single group, as were “Unclear” and “Very unclear,” to avoid violations of expected cell count assumptions. Values of *p *< 0.05 were considered statistically significant.

## RESULTS

### Multidisciplinary consensus panel survey

#### Radiologists

The survey conducted among the 18 radiologists on the LELEX consensus panel (Table 2) revealed a highly experienced group, with 6–36 years of professional experience (mean, 16.8 ± 10.1 years). Half of respondents (50.0%) worked in public and private institutions, and half (50.0%) were affiliated with academic and non-academic entities. Oncologic radiology was the most frequently reported subspecialty (by 38.9%), followed by abdominal radiology (by 33.3%) and internal medicine-related specialties (by 27.8%). In addition, 44.4% of respondents reported involvement in other subspecialties, including thoracic or cardiovascular radiology (16.7%), nuclear medicine (11.1%), neuroradiology or head and neck radiology (11.1%), ultrasound (5.6%), musculoskeletal radiology (5.6%), and breast radiology (5.6%). Notably, several participants practiced across multiple subspecialties, reflecting the multidisciplinary nature of radiologic care in oncology.

**Table 2:** Summary of responses from radiologist members of the LELEX multidisciplinary consensus panel.

Variable	(n = 18)
Years of training, mean ± SD (range)	16.8 ± 10.1 (6-36)
Institution type(s), n (%)	
Public and private	9 (50.0)
Private only	8 (44.4)
Public only	1 (5.6)
Affiliation(s), n (%)	
Academic and non-academic	9 (50.0)
Academic only	6 (33.3)
Non-academic only	3 (16.7)
Subspecialty, n (%)	
Oncologic radiology	7 (38.9)
Abdominal radiology	6 (33.3)
Internal medicine (abdominal and thoracic radiology)	5 (27.8)
Thoracic or cardiovascular radiology	3 (16.7)
Nuclear medicine	2 (11.1)
Neuroradiology or head and neck radiology	2 (11.1)
Ultrasound	1 (5.6)
Musculoskeletal radiology	1 (5.6)
Breast radiology	1 (5.6)
Frequency of oncologic interpretation, n (%)	
Always (almost every day)	14 (77.8)
Very frequently (> 10 days per month)	4 (22.2)
Oncologic imaging exams interpreted per week, mean ± SD (range)	69.4 ± 59.5 (10-250)
Frequency of clarification requests,* n (%)	
Always (almost every day)	4 (22.2)
Very frequently (> 10 days per month)	3 (16.7)
Sometimes (5-10 days per month)	6 (33.3)
Rarely (< 5 days per month)	5 (27.8)
Frequency of structured report use for oncologic exams, n (%)	
Always (almost every day)	4 (22.2)
Very frequently (> 10 days per month)	5 (27.8)
Sometimes (5-10 days per month)	3 (16.7)
Rarely (< 5 days per month)	2 (11.1)
Never	4 (22.2)
Use of a standardized lexicon in oncologic imaging reports, n (%)	
Always (almost every day)	6 (33.3)
Very frequently (> 10 days per month)	6 (33.3)
Sometimes (5-10 days per month)	1 (5.6)
Rarely (< 5 days per month)	1 (5.6)
Never	4 (22.2)
Use of structured reports and a standardized lexicon during training, n (%)	
Always (almost every day)	2 (11.1)
Very frequently (> 10 days per month)	5 (27.8)
Sometimes (5-10 days per month)	3 (16.7)
Rarely (< 5 days per month)	3 (16.7)
Never	5 (27.8)

* Regarding their own oncologic reports or those of other group members.

Radiologists reported a high level of engagement with oncologic imaging: 77.8% interpreted oncologic exams almost daily, with a mean of 69.4 ± 59.5 oncologic imaging exams interpreted per week (range, 10–250 per week). Clarification requests regarding oncologic reports—whether their own or those of colleagues—were also found to be common, with 22.2% of survey participants receiving such inquiries daily and 16.7% receiving them very frequently (defined as more than 10 days per month). Structured reporting was shown to be used with varying frequency, with 22.2% of respondents indicating that they used it daily and 27.8% indicating that they used it very frequently. Standardized lexicons were shown to be relatively well adopted, with one-third of respondents using them daily and another third using them more than 10 days per month. In contrast, the use of structured reports and lexicons during training was less prevalent: only 11.1% reported daily use during their training, and most indicated less frequent use.

#### Non-radiologists

The non-radiologist members of the LELEX consensus panel were also found to be a highly experienced group (Table 3), with a mean of 18.3 ± 10.6 years of professional experience (range, 8–39 years). Most respondents (55.6%) worked in public and private institutions, and a significant proportion were affiliated with academic institutions—either exclusively (44.4%) or in combination with non-academic roles (33.3%). In terms of clinical background, the most common specialties were oncology (33.3%) and radiotherapy (22.2%), followed by surgery (22.2%) and public health (11.1%). One respondent (11.1%), the patient advocate, was affiliated with a nongovernmental organization and actively engaged in patient advocacy through multiple initiatives focused on education, support, and health equity for cancer patients.

**Table 3:** Summary of responses from non-radiologist members of the LELEX multidisciplinary consensus panel.

Variable	(n = 9)
Years of training, mean ± SD (range)	18.3 ± 10.6 (8-39)
Institution type(s), n (%)	
Public and private	5 (55.6)
Private only	3 (33.3)
Public only	1 (11.1)
Affiliation(s), n (%)	
Academic and non-academic	3 (33.3)
Academic only	4 (44.4)
Non-academic only	1 (11.1)
Subspecialty, n (%)	
Oncology	3 (33.3)
Radiotherapy	2 (22.2)
Surgery	2 (22.2)
Public Health	1 (11.1)
Nongovernmental Organization	1 (11.1)
Frequency of reading oncologic reports, n (%)	
Always (almost every day)	7 (77.8)
Rarely (< 5 days per month)	2 (22.2)
Frequency of consulting a radiologist for clarification on a radiology report, n (%)	
Always (almost every day)	2 (22.2)
Very frequently (> 10 days per month)	3 (33.3)
Sometimes (5-10 days per month)	2 (22.2)
Rarely (< 5 days per month)	1 (11.1)
Never	1 (11.1)
Frequency of reading structured reports	
from oncologic imaging exams, n (%)	
Always (almost every day)	1 (11.1)
Very frequently (> 10 days per month)	1 (11.1)
Sometimes (5-10 days per month)	4 (44.4)
Rarely (< 5 days per month)	1 (11.1)
Never	2 (22.2)
Frequency of reading standardized lexicon from oncologic imaging exams, n (%)	
Always (almost every day)	0 (0)
Very frequently (> 10 days per month)	2 (22.2)
Sometimes (5-10 days per month)	2 (22.2)
Rarely (< 5 days per month)	2 (22.2)
Never	3 (33.3)

Most non-radiologists were engaged closely with oncologic imaging in their practice: 77.8% reported reading oncologic imaging reports almost daily. With regard to seeking clarification on radiology reports, 22.2% reported that they did so daily, whereas one-third (33.3%) indicated that they did so more than 10 times per month. Survey results indicated that structured reports are commonly encountered by the non-radiologists, though not as consistently as unstructured reports. Overall, 44.4% of respondents reported reading structured reports occasionally (5–10 times per month), whereas 22.2% reported never reading them. The use of standardized lexicons was even less common—no respondents reported daily use, and one-third (33.3%) indicated they never used them.

#### Comparison between the traditional free-text format and structured reporting with a standardized lexicon

In the evaluation of a free-text report in comparison with a structured report employing a standardized lexicon (Report 1 and Report 2, respectively), the radiologists on the panel (n = 18) showed a strong preference for Report 2 across all assessed domains (Table 4). A significant majority rated Report 2 as “Very clear” and “Very precise” (83.3% and 72.2%, respectively), whereas Report 1 received markedly lower ratings for clarity and precision. Likewise, 77.8% of radiologists were “Very satisfied” with Report 2, compared with none for Report 1. Statistical analysis confirmed these differences were significant across all three parameters: clarity rating (*p *< 0.001), precision rating (*p* < 0.001), and satisfaction level (*p *< 0.001). Report 2 was the top choice for both clarity in describing imaging findings and usefulness in guiding clinical decision-making, with 88.9% of respondents selecting it as the most useful report; the remaining respondents indicated that the two reports were equally useful.

**Table 4:** Distribution of responses from radiologists and non-radiologists on the LELEX multidisciplinary consensus panel regarding their impressions of report 1 (traditional free-text format) and report 2 (structured report with standardized lexicon).

Category	Response	Report 1	Report 2	P
		n (%)	n (%)	
Radiologists				
	Very clear	1 (5.6)	15 (83.3)	
	Clear	6 (33.3)	2 (11.1)	
Clarity rating	Neutral	3 (16.7)	1 (5.6)	0.0001
	Unclear	7 (38.9)	0 (0.0)	
	Very Unclear	1 (5.6)	0 (0.0)	
	Very precise	1 (5.6)	13 (72.2)	
	Somewhat precise	4 (22.2)	4 (22.2)	
Precision rating	Neutral	3 (16.7)	1 (5.6)	0.0003
	Somewhat imprecise	7 (38.9)	0 (0.0)	
	Very imprecise	3 (16.7)	0 (0.0)	
	Very satisfied	0 (0.0)	14 (77.8)	
	Somewhat satisfied	5 (27.8)	3 (16.7)	
Satisfaction level	Neutral	2 (11.1)	1 (5.6)	0.00003
	Somewhat dissatisfied	8 (44.4)	0 (0.0)	
	Very dissatisfied	3 (16.7)	0 (0.0)	
Non-radiologists				
	Very clear	0 (0.0)	5 (55.6)	
	Clear	5 (55.6)	4 (44.4)	
Clarity rating	Neutral	2 (22.2)	0 (0.0)	0.076
	Unclear	2 (22.2)	0 (0.0)	
	Very Unclear	0 (0.0)	0 (0.0)	
	Very precise	1 (11.1)	3 (33.3)	
	Somewhat precise	3 (33.3)	6 (66.7)	
Precision rating	Neutral	2 (22.2)	0 (0.0)	0.031
	Somewhat imprecise	3 (33.3)	0 (0.0)	
	Very imprecise	0 (0.0)	0 (0.0)	
	Very satisfied	0 (0.0)	4 (44.4)	
	Somewhat satisfied	5 (55.6)	5 (55.6)	
Satisfaction level	Neutral	2 (22.2)	0 (0.0)	0.076
	Somewhat dissatisfied	2 (22.2)	0 (0.0)	
	Very dissatisfied	0 (0.0)	0 (0.0)	

Among the non-radiologists on the panel (n = 9), Report 2 was also rated more favorably (Table 4), though the differences were not statistically significant for clarity (*p *= 0.076) or satisfaction (*p *= 0.076). Nonetheless, 55.6% of respondents rated Report 2 as “Very clear” and 44.4% indicated they were “Very satisfied” with it, whereas no respondents selected >those options for Report 1. The precision ratings favored Report 2 significantly (*p *= 0.031), with 33.3% rating it “Very precise.” Among non-radiologists, 88.9% selected Report 2 as better for describing imaging results and guiding clinical decisions, whereas the remainder considered the two reports equally effective.

### Oncologic lexicon

On the basis of the methodology adopted by the LELEX multidisciplinary consensus panel, consensus was reached for all core components of the proposed lexicon and reporting system (Table 1 and Supplement 3). The consensus methodology was applied to four essential elements: the diagnostic certainty lexicon, lesion numbering, comparison terminology, and guidance for reporting representative lesions. Each element was carefully defined and refined to promote clarity, consistency, and clinical relevance. For example, diagnostic certainty terms were standardized and linked to estimated probabilities as follows: “Consistent with” (> 90%); “Suggestive of” (~ 75%); “Possible” (~ 50%); and “Less likely” (< 25%).

**Supplement 3.** Portuguese translation of the LELEX oncologic lexicon.

**1 - Léxico de certeza**	
**Termo de certeza**	**Estimativa de probabilidade**
Compatível com	> 90%
Sugestivo de	~ 75%
Possível	~ 50%
Pouco provável	< 25%
**2 Léxico de número de lesões**	
**Termo de número**	**Número estimado**
Poucos (as)	1–5
Múltiplos (as)	> 6
**3 Comparação – interpretação subjetiva**	
**Termo comparativo**	
Desaparecimento	
Redução significativa	
Redução	
Discreta redução	
Inalterado (a)	
Discreto aumento	
Aumento	
Novo (a)	
**4 Lesões representativas (2 por órgão)**	
Localização, [] × []* cm, anteriormente [] × []* cm	
* 2 eixos ortogonais no plano axial	
**5 Exemplo**	
Aumento dos poucos nódulos hepáticos, compatíveis com metástases, por exemplo:	
Segmento 2, 2.5 × 2.0 cm, previamente 1.5 × 1.0 cm	
Segmento 5, 1.8 × 1.0 cm, previamente 0.5 × 0.5 cm	

The panel members agreed that if an imaging finding is clearly diagnostic—as in the case of a simple cyst, classic hemangioma, or previously confirmed metastasis—the use of the certainty lexicon (e.g., “Suggestive of” or “Possible”) is not necessary. In these cases, straightforward language is preferred to avoid redundancy and enhance clarity. For example, “Unchanged multiple liver metastases”, followed by measurements, is sufficient when there is no diagnostic uncertainty. In addition, clearly benign incidental findings, such as renal or hepatic cysts, do not require detailed description or comparison with prior studies. A brief mention— for example, “renal cysts”—is appropriate. This approach minimizes overinterpretation and helps maintain focus on clinically relevant findings.

The panel also agreed on terms to describe the lesion count, recommending “Few” for 1–5 lesions and “Multiple” for ≥ 6, providing a consistent framework to quantify disease burden. For longitudinal comparisons, the panel endorsed standardized descriptors such as “new,” “increased,” “slightly increased,” “unchanged,” “slightly decreased,” “markedly decreased,” and “resolved,” helping streamline interpretation of treatment response or disease progression. The use of the term “markedly increased” is discouraged, because the descriptor “increased” alone sufficiently conveys the necessary information for treatment planning, and the qualifier “markedly” may cause undue anxiety when interpreted by patients. Lastly, consensus was reached on reporting two representative lesions per organ site, including lesion location, size in two axial perpendicular dimensions, and prior measurements when applicable.

### Structured reporting system general oncologic imaging assessment

The LELEX structured reporting system, agreed upon by the panel, outlines key elements to include in a general oncologic imaging report (Supplement 4). These elements begin with the identification of the imaging modality, body region, and date of the exam, followed by relevant clinical information. The technique section should detail the use of intravenous and oral contrast, the acquisition method, and post-processing parameters. When applicable, the report should include a comparison with prior imaging studies, specifying the modality and date. The findings section should be organized by organ system. When no relevant abnormalities are found, brief descriptors such as unremarkable or normal are recommended. Clearly benign incidental findings may be described at the radiologist’s discretion; if included, the description should be concise (e.g., “simple renal cyst”) and avoid unnecessary detail. Lastly, the impression should provide a succinct summary of key findings, including comparisons with the prior imaging available.

**Supplement 4.** Portuguese translation of the LELEX structured reporting system.

**[Data] TOMOGRAFIA DE TÓRAX, ABDOME E PELVE DADOS CLÍNICOS: []**	
**TÉCNICA:**	
Meio de contraste intravenoso: com / sem contraste	
Meio de contraste oral: não / sim	
Aquisição: axial de TC de tórax, abdome e pelve	
**COMPARAÇÃO:** XXX (Modalidade e data – Estudos usados para comparação direta)	
**ACHADOS:**	
PULMÕES/VIAS AÉREAS:	Sem alterações relevantes.
PLEURA/PERICÁRDIO:	Ausência de derrame.
MEDIASTINO/ LINFONODOS TORÁCICOS:	Ausência de linfonodopatia.
HEPATOBILIAR:	Sem alterações relevantes.
BAÇO:	Sem alterações relevantes.
PÂNCREAS:	Sem alterações relevantes.
ADRENAIS:	Sem alterações relevantes.
RINS:	Sem alterações relevantes.
LINFONODOS	
ABDOMINO-PÉLVICOS:	Ausência de linfonodopatia
ÓRGÃOS PÉLVICOS:	Sem alterações relevantes.
PERITÔNIO/MESENTÉRIO/ INTESTINO:	Sem alterações relevantes.
OSSO/PARTES MOLES:	Ausência de lesões suspeitas
OUTROS:	Nenhum.
**IMPRESSÃO:**		Em comparação com XXX:	
Termo de certeza	Estimativa de probabilidade
Compatível com	> 90%
Sugestivo de	~ 75%
Possível	~ 50%
Pouco provável	< 25%

## DISCUSSION

This study describes the LELEX initiative, a multidisciplinary effort to establish an agreed-upon standardized lexicon and structured reporting system to enhance the clarity and consistency of oncologic imaging communication. Building on established frameworks and adapted to real-world clinical needs, the LELEX system was designed to enhance clarity, consistency, and clinical relevance in the reporting of general oncologic follow-up imaging. The initiative addresses a critical gap in routine practice by promoting a unified language that can be applied across tumor types and specialties to facilitate better communication and decision-making across a wide array of multidisciplinary oncology teams.

Survey results from the LELEX steering committee confirmed the need for improved standardization in radiologic reporting. Radiologists and non-radiologists, representing diverse oncologic disciplines, acknowledged the limitations of traditional free-text reports and supported the adoption of a structured format and lexicon-based communication. Radiologists found the structured report significantly clearer, more precise, and more satisfactory compared with the freetext version. Non-radiologists expressed similar preferences, particularly for clinical utility. It is noteworthy that the standardized lexicon and report structure evaluated in the survey were based on prior evidence from a certainty lexicon^**(^13^)**^ and an oncologic response lexicon^**(^14^)**^, which were carefully adapted to reflect local workflows and language.

Our findings are consistent with those of previous studies demonstrating that structured reports improve clarity, confidence in interpretation, and interdisciplinary communication^**(^1,3,5,6^)**^. Although several established templates exist for specific tumor types (e.g., BI-RADS, LI-RADS, and PI-RADS) and RECIST standardized treatment response assessment criteria are widely used (mainly in clinical trials), these tools do not address the broader need for a standardized, structured format for general oncologic imaging baseline and follow-up assessment applicable to real-world workflows and across cancer types. The LELEX initiative builds upon prior efforts by introducing a system designed for broader use in clinical practice—complementing, rather than competing with, existing organ-specific reporting systems. It aligns with global efforts to streamline oncologic imaging assessment through scalable, consistent frameworks that enhance multidisciplinary care.

This study has some limitations. First, although the panel was multidisciplinary and nationally representative, the sample size (i.e., number of panel members) was relatively small and may not reflect the reality in all practice settings. In addition, the evaluation of report preferences was based on survey responses and subjective metrics, which, although informative, may be influenced by familiarity with structured reporting or local practice patterns. Furthermore, our study did not include assessment of implementation feasibility and user experience in routine clinical workflows, which will require further prospective studies.

While the initial phase of the LELEX initiative focuses on general baseline and follow-up oncologic imaging assessment, future directions include developing specialized lexicons and structured reporting templates tailored to specific tumor types—such as surgical and locoregional evaluation of liver metastases, as well as evaluation of rectal, prostate, lung, and gynecologic malignancies—across various clinical scenarios, including baseline staging, restaging, and surveillance. Expansion into additional subspecialties, such as neuroradiology, is also anticipated. In parallel, ongoing studies aim to evaluate the real-world impact of the LELEX structured reporting template and standardized lexicon on clinical workflows, decision-making, and patient outcomes. The long-term vision is to support the widespread adoption of standardized, structured oncologic imaging communication that aligns with the evolving needs of modern cancer care and multidisciplinary collaboration.

In conclusion, the LELEX initiative successfully established a consensus-based lexicon and structured report for general oncologic imaging with the input and approval of radiologists and non-radiologists from diverse oncologic specialties and practice settings. By standardizing terminology and report structure, the initiative aims to enhance communication, reduce ambiguity, and improve clinical integration of imaging findings. Our results support the need for further research to assess and validate the clinical impact of the LELEX structured reporting system for general oncologic imaging across a broad spectrum of oncologic care settings.

## Data Availability

The research data are available upon request.
